# Assessing the anti-fungal efficiency of filters coated with zinc oxide nanoparticles

**DOI:** 10.1098/rsos.161032

**Published:** 2017-05-03

**Authors:** Stephen Decelis, Davide Sardella, Thomas Triganza, Jean-Pierre Brincat, Ruben Gatt, Vasilis P. Valdramidis

**Affiliations:** 1 Mycology laboratory, Mater Dei Hospital, Msida, Malta; 2 Centre for Molecular Medicine and Biobanking, University of Malta, Msida, Malta; 3 Department of Food Studies and Environmental Health, Faculty of Health Sciences, University of Malta, Msida, Malta; 4 Metamaterials Unit, Faculty of Science, University of Malta, Msida, Malta

**Keywords:** filters, zinc oxide, nanoparticles, *Penicillium expansum*, *Rhizopus stolonifer*, inhibition

## Abstract

Air filters support fungal growth, leading to generation of conidia and volatile organic compounds, causing allergies, infections and food spoilage. Filters that inhibit fungi are therefore necessary. Zinc oxide (ZnO) nanoparticles have anti-fungal properties and therefore are good candidates for inhibiting growth. Two concentrations (0.012 M and 0.12 M) were used to coat two types of filters (melt-blown and needle-punched) for three different periods (0.5, 5 and 50 min). *Rhizopus stolonifer* and *Penicillium expansum* isolated from spoiled pears were used as test organisms. Conidial suspensions of 10^5^ to 10^3^ spores ml^−1^ were prepared in Sabouraud dextrose agar at 50°C, and a modified slide-culture technique was used to test the anti-fungal properties of the filters. *Penicillium expansum* was the more sensitive organism, with inhibition at 0.012 M at only 0.5 min coating time on the needle-punched filter. The longer the coating time, the more effective inhibition was for both organisms. Furthermore, it was also determined that the coating process had only a slight effect on the Young's Moduli of the needle-punched filters, while the Young's Moduli of the melt-blown filters is more susceptible to the coating method. This work contributes to the assessment of the efficacy of filter coating with ZnO nanopaticles aimed at inhibiting fungal growth.

## 1. Introduction

Air is a vehicle for bio aerosols, which include among other entities, fungal spores. These fungal entities are highly resistant to adverse environmental conditions. Fungal spores are undesired air particles in particular environments as these can cause problems such as allergies, infections and food spoilage [1–3]. Air filters are therefore essential in various settings, including healthcare facilities, food manufacturing facilities and other buildings, as it must be ensured that the desired air quality is achieved in these settings. There are various types of air filters, depending on the air quality that is required. These range from high efficiency particulate air (HEPA) filters, found in specific hospital settings, where patients at high risk of infection are treated [2], to simple filters that trap larger particles in domestic and vehicle air conditioning units. Other examples include the use of polyurethane foams, nanofibre mats and other fibre filters [4]. Complex buildings, such as hotels and office blocks make use of heating, ventilating and air conditioning (HVAC) systems, which also incorporate air filters. Food manufacturing and processing units are another example of specialized facilities, where air quality in terms of bio aerosols is essential [3].

Air filters are usually disposable, and are designed to filter a fixed volume of air. Therefore, such air filtration equipment needs to be changed regularly to ensure effectiveness. The material used for filters is inert; however, trapped material may include organic particles and microorganisms. Fungal spores and conidia trapped in a filter are not killed and may encounter the ideal environmental conditions, which include optimal moisture, temperature and nutrients, to germinate and form a mycelium on the filter itself. This can lead to the generation of conidia that may be dispersed in the environment [5,6]. Thus a filter may be responsible for seeding the environment with microorganisms it should be filtering out. Apart from allergies, infections and food spoilage, such growth may also release volatile organic compounds such as acetone, carbonyl sulfide, ketones, aldehydes and alcohols [1,5]. These compounds are known to be irritants to humans and chemical contaminants in food products. It is therefore important to inhibit the growth of fungi on filters.

A number of methods can be used to inactivate spores and conidia trapped in a filter, in order to prevent mycelium from developing. These range from UV irradiation (with or without a photocatalyst), fumigation, and silver, copper and zinc oxide nanoparticle coating among others [4]. Nanocoating seems to be the method with the least disadvantages, as for example UV radiation may have penetration problems, and fumigation may lead to toxic vapour accumulation.

Zinc oxide nanoparticles are very important due to their utilization in gas sensors, biosensors, cosmetics, drug-delivery systems, etc. [7]. ZnO nanoparticles were shown to possess antimicrobial properties [8–10]. Mechanisms of action have been proposed for bacterial cells, but these can also be considered for fungal cells. These properties are thought to be both chemical and physical. Chemical antimicrobial properties are thought to be due to the ability of these particles to generate reactive oxygen species (ROS), which would then result in the formation of hydrogen peroxide. This would then interact with the cell membrane of the microorganism, leading to oxidation of the lipids, rendering the membrane dysfunctional [11,12]. Physical antimicrobial properties are thought to be brought about by physical blockage of transport channels in the membrane, and direct membrane damage due to the shape and size of these particles [12].

*Rhizopus* and *Penicillium* spp. are two important fruit contaminants. *Rhizopus stolonifer* is a cosmopolitan fungus located mainly in warehouses on the conditioning material and on stored fruits, especially strawberry, apple, plum, peach and pear [13,14]. *Rhizopus stolonifer* is responsible for Rhizopus rot, sometimes also referred as ‘soft rot’ or ‘whiskers rot’, on peaches, plums and nectarines [15]. Rhizopus rot, affects mainly injured, freeze-damaged and overripe fruits [16]. *Rhizopus stolonifer* is one of the most important postharvest stone fruit decay agents in Europe [17]. There are also reports of Rhizopus rot on jackfruit [18], on papaya [19] and on sweet potato [20]. This fungus is a very aggressive wound parasite which is difficult to control once fruit ripens [21]. Rhizopus rot is no longer efficiently controlled by registered fungicides [17] and, if the storage temperature is higher than 5°C, the rot spreads rapidly from the infected to adjacent fruits. Dormant spores of *R. stolonifer*, isolated from peach fruit, were found to be resistant to chilling temperatures as low as −1°C [22]. A low temperature strain of *R. stolonifer* was also observed in the laboratory by Sommer [15]. Recent reports of cold-resistant *R. stolonifer* strains have also been reported [23] where this fungus is the main causative agent of strawberry leak and it is able to grow and colonize this fruit at about 3°C. On the other hand, *P. expansum* is responsible for blue mould, the most important postharvest disease of apples and pears. Before the advent of controlled-atmosphere facilities, it accounted for as much as 90% of the total postharvest losses of apples [16]. The incidence of blue mould is often less than 1% in modern storage facilities, but it is still the most common postharvest decay in pome fruit [16]. *Penicillium expansum* is also normally found in stone fruit only after a week in storage at 0°C [15]. Peach fruits are easily infected by *P. expansum*, causing blue mould rot in the fruit and leading to considerable postharvest losses [24]. *Penicillium expansum*'s infection can also occur on ripe and overripe oranges under particular temperature conditions [25].

Studies on the anti-fungal activity of ZnO nanoparticles have been carried out against fruit spoilage fungi such as *Botrytis cinerea*, *P. expansum*, *R. stolonifer* and *Fusarium graminearum* [8,9]. It was shown that such particles inhibited development of conidia, conidiophores showed distortion and hyphae were deformed [9]. Therefore, testing nanoparticle-coated filters for inhibition of food spoilage organisms, such as *P. expansum* and *R.stolonifer*, may potentially shed light on how to better control specific environments against these organisms, particularly in food storage facilities.

The aim of this study was to assess the anti-fungal efficiency of melt-blown and needle-punched filters coated with ZnO nanoparticle solutions. The most effective coating conditions, i.e. nanoparticle concentration and coating time, for the inhibition of fungal conidial suspensions, *P. expansum* and *R. stolonifer,* previously isolated from pome fruits, are identified.

## 2. Material and methods

### 2.1. Filters

EN 779 standard filters, particulate air filters for general ventilation, were used. Two types were used: needle-punched HS-Alpha Pak (efficiency 40–60%, Δ*P* = 40–50 Pa) made of synthetic fibre fleece and chosen for its maximum filtration abilities at minimal dimensions, henceforth referred to as needle-punched filter, and melt-blown HS-Beta Pak (efficiency 65–95%, Δ*P* = 90–150 Pa) made of fibreglass paper and chosen for its properties as a robust dust filter, henceforth referred to as melt-blown filter. Filters were manufactured by Luftfilterbau GMBH (Kiel, Germany). The filter samples (each cut to a size of 10 mm by 10 mm) that were used for the anti-fungal tests (see below) were autoclaved at 135°C for 3 min to eliminate any microbial contaminants that might be present. On the other hand, the filter samples which were used to conduct the mechanical tests (each cut to a size of 20 mm by 80 mm) were not subjected to autoclaving.

### 2.2. Nanoparticle coating of filters

Commercially available ZnO nanoparticles (Sigma Aldrich, USA) were used throughout this study. ZnO nanoparticle suspensions were prepared by mixing the ZnO nanoparticles (in powder form) with propan-2-ol. This suspension was then sonicated for 30 min at 60 Hz. Two different concentrations of ZnO nanoparticles suspensions were prepared, namely 0.012 M and 0.12 M (within the range of previously reported studies on the anti-fungal properties of ZnO [9]) in solutions of propan-2-ol.

The filters were then coated by immersion in a nanoparticle suspension. More specifically, for each of the filters tested (needle-punched and melt-blown) nine different samples were prepared in triplicate. For each of the nine samples prepared, different immersion times and/or concentration of the nanoparticles suspension were used as detailed in table 1. The treated filters were left to dry overnight in a sterile laminar flow hood in order to be dried before further use.

**Table 1.** Sample coating preparations. Note that X in sample ID can be ‘N’ indicating the needle-punched filters or ‘M’ indicating the melt-blown filters (negative refers to propan-2-ol samples). n.a., not available*.*

sample ID	immersion time (minutes)	concentration of nanoparticles (molar)
X_0_	0 (positive control)	n.a.
X_1_	0.5	0 (negative control)
X_2_	5	0 (negative control)
X_3_	50	0 (negative control)
X_4_	0.5	0.012
X_5_	5	0.012
X_6_	50	0.012
X_7_	0.5	0.12
X_8_	5	0.12

### 2.3. Scanning electron microscopy analysis

Micrographs of the filter at a magnification of 500 were captured using a Carl Zeiss Merlin Field Emission scanning electron microscope (SEM), with an extra high tension (EHT) of 2.20 kV and a working distance (WD) of 5.0 mm. The filters were mounted on an aluminium block using carbon tape (as conductive substrates) based on commonly used approaches.

### 2.4. Preparation of conidial suspensions

Two mould fungi were selected for testing: *P. expansum* and *R. stolonifer*. These two organisms were selected because of their important impact in postharvest pathology, as previously described. Fungal spores were harvested from 5-day-old malt extract agar (MEA) (Biolife, Italy) Petri dish cultures by adding 10 ml of a 0.05% Tween-80 solution and by scraping off the plates' surfaces with a sterile bent rod. The resulting suspensions were aseptically filtered through a four-layer sterile gauze to remove any mycelial contamination. The concentrations of such ‘dormant’ spores' suspensions were determined with a Neubauer counting chamber in order to prepare the dilutions required for the testing of the filters.

For *P. expansum*, a suspension of 1.73 × 10^6^ conidia ml^−1^ was achieved. This was serially diluted in sterile distilled water to achieve 10-fold dilutions down to 1.73 × 10^3^ conidia ml^−1^. For *R. stolonifer*, a conidium suspension of 4.12 × 10^5^ conidia ml^−1^ was achieved. This was serially diluted in sterile distilled water to achieve 10-fold dilutions down to 4.12 × 10^2^ conidia ml^−1^.

### 2.5. Fungal inhibition of coated filters

A modification of the slide-culture method [26] was used to test the nanoparticles coated filters for growth inhibition. Sabouraud dextrose agar (SDA) (Biolife, Italy) was prepared to soak the coated filters in order to hasten the fungal growth onto their surfaces. This would represent a worst case scenario for filters to support fungal growth. The conidial suspensions were used to inoculate the SDA tempered down to 50°C. The following final concentrations were obtained: for *P. expansum*, 1.73 × 10^4^, ×10^3^, and ×10^2^ conidia ml^−1^; for *R. stolonifer*, 4.21 × 10^3^, ×10^2^, and ×10^1^ conidia ml^−1^.

The cut filters were mounted on a sterile slide. The slide was then set up onto a bent glass rod or sterile bent plastic straw in a 90 mm sterile glass Petri dish. In total, 5 ml of sterile distilled water was added to the bottom of the Petri dish to keep the humidity high and constant. The filters were then soaked from both sides with the agar suspension containing the conidia using a sterile plastic pipette and sterile plastic forceps. For each suspension, one type of nanoparticle-coated filter produced earlier was tested and one non-coated filter was also tested as positive control. The propan-2-ol control filters were also tested as negative controls. In total, 25 ml from each remaining inoculated agar suspension were then used to fill one sterile 90 mm plastic Petri dish that was incubated together with the other ones. This control was used to make sure that conidia remained viable when preparing the suspensions at 50°C. The set-ups were allowed to set for a few minutes and then incubated at 25°C for 5 days. After incubation was complete, tests were compared with controls to see where growth was inhibited in nanoparticle-coated filters. Experiments were reported as binary responses (for similar approaches refer to Tassou *et al*. [27].

### 2.6. Mechanical properties of filters

The mechanical properties, namely the Young's modulus (which describes the relation between stress and strain) and Poisson's ratio (which describes the change in the transverse strain in response to an axial strain), of the different samples of coated filters were measured using a tensile loading machine (Testometric, UK) having a 100 kgF load cell (S/N 31931), equipped with a duly calibrated camera video-extensometer (Messphysik, Germany). Samples measuring 20 mm by 80 mm were coated as explained above. For each immersion time/nanoparticle solution concentration used (as detailed in table 1 above), four repeats were performed. The Young's modulus was measured as the slope of a stress–strain graph.

In order to measure the Poisson's ratio, axial and transverse strains of the measurements were taken for the length and width of the filters samples, which were appropriately marked for the Messphysik pattern recognition software. For each filter sample, three transverse lengths and one axial length were recorded. As much as possible, the measurements were taken from the centre of the specimen in order to reduce any edge effects. Experiments were run up to 1% axial strain. The Poisson's ratio was then measured as the negative slope of a transverse strain–axial strain graph.

## 3. Results

### 3.1. Coating of foams

**Figure 1.**
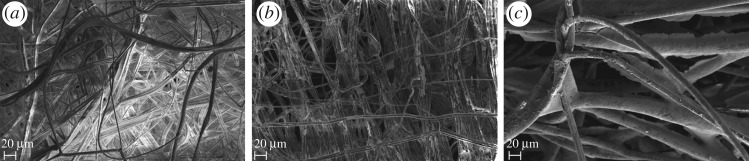
SEM images of the coated filters. (*a*) Melt-blown filter immersed in 0.12 M ZnO nanoparticles solution for 5 min, (*b*) melt-blown filter immersed in 0.12 M ZnO nanoparticles solution for 50 min and (*c*) needle-punched filter immersed in 0.12 M ZnO nanoparticles solution for 0.5 min.

A visual analysis of the SEM images (figure 1) indicates that, as expected, increasing the immersion time increases the thickness of the nanoparticle coating on the filter fibres. It is also evident that the needle-punched filters are more heavily coated than the melt-blown equivalent. In fact, using the same nanoparticle suspension, the coat on the needle-punched filters after 0.5 min of immersion (figure 1*c*) is much thicker than the coat on the melt-blown filter after 50 min of immersion (figure 1*b*).

### 3.2. Anti-fungal activity of coated filters

**Figure 2.**
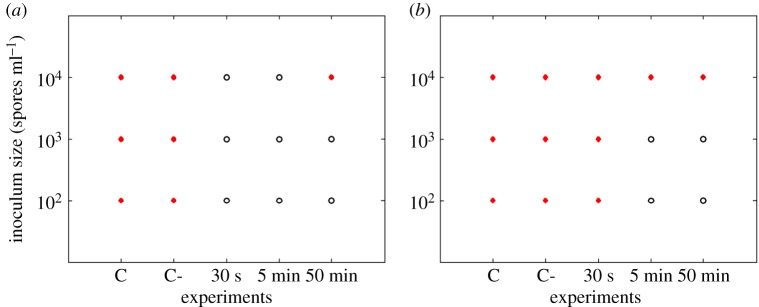
*Penicillium expansum* responses (recovery: closed circles, no recovery: open circles) on needle-punched (*a*) and melt-blown (*b*) filters coated with 0.012 M ZnO. C refers to control samples and C- to control samples soaked in propan-2-ol solution.

**Figure 3.**
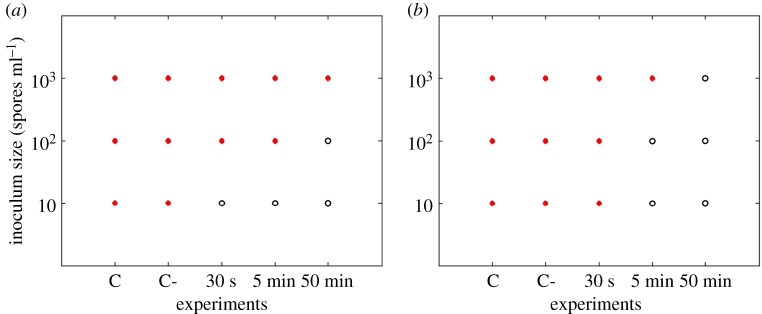
*Rhizopus stolonifer* responses (recovery: closed circles, no recovery: open circles) on needle-punched (*a*) and melt-blown (*b*) filters coated with 0.012 M ZnO. C refers to control samples and C- to control samples soaked in propan-2-ol solution.

**Figure 4.**
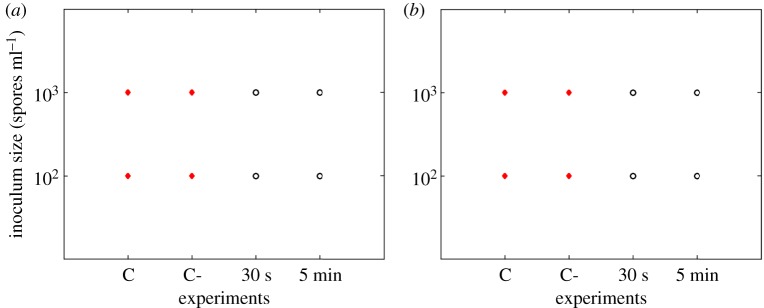
*Penicillium expansum* responses (recovery: closed circles, no recovery: open circles) on needle-punched (*a*) and melt-blown (*b*) filters coated with 0.12 M ZnO. C refers to control samples and C- to control samples soaked in propan-2-ol solution.

**Figure 5.**
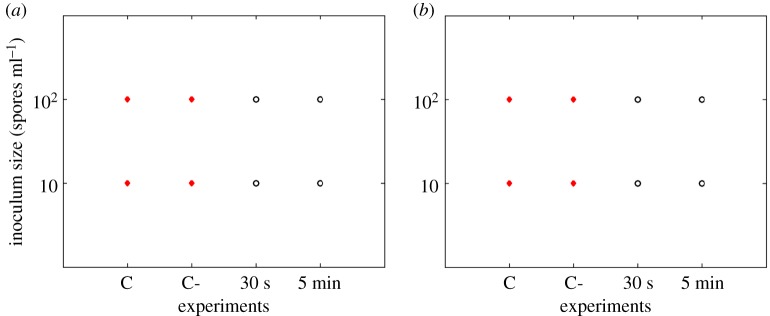
*Rhizopus stolonifer* responses (recovery: closed circles, no recovery: open circles) on needle-punched (*a*) and melt-blown (*b*) filters coated with 0.12 M ZnO. C refers to control samples and C- to control samples soaked in propan-2-ol solution.

Results show that *P. expansum* was the most inhibited on the 0.012 M ZnO coated filters and could even be inhibited when filters were coated for only 30 s in the case of needle-punched filters (figure 2). *Rhizopus stolonifer* showed less sensitivity when compared with *P. expansum* (figure 3). The longer the coating time, the more effective the inhibition for all the tested fungi. The 0.12 M concentration of ZnO inhibited all the studied fungi even for the shortest coating time of 30 s (figures 4 and 5).

### 3.3. Mechanical properties of coated filters

**Figure 6.**
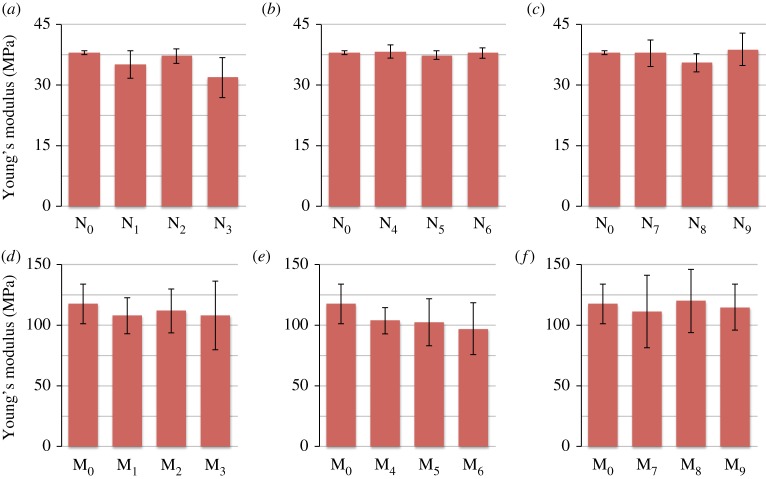
Young's modulus measurements for (*a*) needle-punched filters controls (*b*) needle-punched filters immersed in 0.012 M nanoparticles suspension, (*c*) needle-punched filters immersed in 0.12 M nanoparticles suspension, (*d*) melt-blown filters controls (*e*) melt-blown filters immersed in 0.012 M nanoparticles suspension, (*f*) melt-blown filters immersed in 0.12 M nanoparticles suspension.

From figure 6, it is clear that the measurements of the Young's modulus for the needle-punched filters are much less variable than those for the melt-blown filters, which means that the four needle-punched samples tested are much more alike than the four melt-blown samples tested. Furthermore, the needle-punched filters have a lower Young's modulus than melt-blown filters.

When comparing the dry controls to the wet controls, it is apparent that the needle-punched filters are affected more by longer immersion times than the melt-blown filters. However, when coated with ZnO nanoparticles, the Young's modulus of the melt-blown filters changes more than that of the needle-punched filters. This is indicated by the higher variability in the measurements obtained for the coated melt-blown filters.

The results obtained for the Young's moduli of the filters indicate that while the solvent causes the Young's modulus to decrease, as observed in the wet control, the presence of the nanoparticles causes the modulus to increase, which explains the higher moduli observed at high nanoparticle concentration. Solvent effect and nanoparticle effect on the filters probably act in an opposing manner, yielding the results obtained.

**Figure 7.**
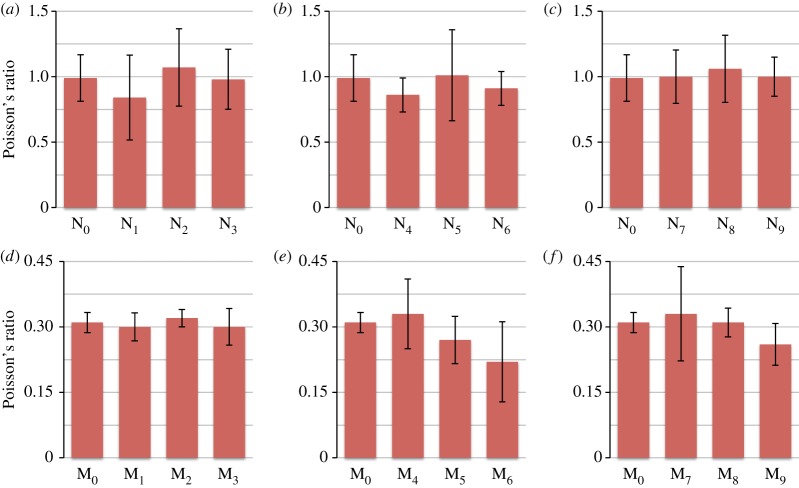
Poisson's ratio measurements for (*a*) needle-punched filters controls, (*b*) needle-punched filters immersed in 0.012 M nanoparticles suspension, (*c*) needle-punched filters immersed in 0.12 M nanoparticles suspension, (*d*) melt-blown filters controls, (*e*) melt-blown filters immersed in 0.012 M nanoparticles suspension, (*f*) melt-blown filters immersed in 0.12 M nanoparticles suspension.

When comparing the results for the Poisson's ratio measurements, it is evident from figure 7 that the Poisson's ratio of the needle-punched filters is higher (close to 1) than those of the melt-blown filters (close to 0.3). Overall, none of the treatments performed on the two types of filters seemed to have a large effect on the Poisson's ratio; this indicates that the coating process does not alter the mechanical properties of the filters. The small changes in Poisson's ratio may be attributed to a change in the way the fibres behave when subjected to a load (a change in deformation mechanism), due to the presence of the nanoparticles. Furthermore, the high variability in the results, particularly for the needle-punched filters, can be attributed to the random arrangement of fibres.

## 4. Discussion

The anti-fungal properties of ZnO nanoparticles were demonstrated quite clearly in this study as both the fungi were sensitive to the nanoparticles' effect. According to the obtained results, *P. expansum* seemed to recover from the inhibitory effect of ZnO more slowly than *R. stolonifer*. This was shown to be the case at the highest inoculum densities where *Rhizopus* still managed to grow while *Penicillium* failed. The lower recovery ability of *Penicillium* could be due to the difference in the growth dynamics between these two fungi because *Rhizopus* is well known to show a higher aggressiveness even when grown in non-optimal conditions [28,29].

Damage caused by nanoparticles depends on size [30] and shape. Nanoparticles vary in shape, and these can be triangular, rod shaped or spherical [11,31,32]. This affects the surface area to volume ratio, and also the ability to cause physical damage to cells [12]. Because of the size, a relatively high surface area to volume ratio is achieved and presumably this is the most important property leading to the antimicrobial effects. Research has focused mainly on bactericidal mechanisms. Fungicidal mechanisms have been much less studied. Proposed fungicidal mechanisms of nanoparticles are extrapolated from mechanisms proposed for bacteria and include interaction with thiol groups of vital enzymes leading to enzyme inactivation [33], and killing by oxidative stress [11]. ROS was, however, demonstrated to cause membrane and cell wall damage.

Inhibition of growth seemed to be less on melt-blown filters rather than the needle-punched ones. This may be explained by the amount of nanoparticles that adhere to the fibres of the filter. In fact, from figure 1 (electron micrographs showing the coating on the filters), it is clear that ZnO nanoparticles adhere much more to the needle-punched filters rather than to the melt-blown ones. This means that the spores in contact with the coated needle-punched filters found a harsher environment (higher amount of ZnO nanoparticles) when compared with the spores in contact with the coated melt-blown filters. The identification of such commercial filters that can be used for coating with nanoparticles, while preventing the growth of fungi without affecting the mechanical properties of the filter, are of importance for novel industrial applications.

ZnO nanoparticles may therefore be good candidates for coating various filters needed to remove microorganisms from the air. Nanoparticle-coated filters may be a good investment, as these can potentially have a longer shelf life once they are installed due to the fact that these will not suffer contamination problems. It is therefore highly desirable to explore the potential of such filters further.

## Supplementary Material

Assessing the Anti-fungal Efficiency of Filters Coated with Zinc Oxide Nanoparticles

## Data accessibility

Data are available at the Dryad Digital Repository. http://dx.doi.org/10.5061/dryad.15358 [34].
