# Supplementary material for: Assessing the anti-fungal efficiency of filters coated with zinc oxide nanoparticles
Source: R Soc Open Sci. 2017 May 3;4(5):161032. doi: 10.1098/rsos.161032 (PMC5451796; doi:10.1098/rsos.161032)
Supplement: Assessing the Anti-fungal Efficiency of Filters Coated with Zinc Oxide Nanoparticles [file rsos161032supp1.doc]

**Supplementary Information**

**Assessing the Anti-fungal Efficiency of Filters Coated with Zinc Oxide Nanoparticles**

Stephen Decelis^1^, Davide Sardella*^2^*, Thomas Triganza*^2^*, Jean-Pierre Brincat^3^, Ruben Gatt*^3^**, Vasilis P. Valdramidis^1^*

*^1^Mycology laboratory, Mater Dei Hospital, Msida, Malta*

*^2^Department of Food Studies and Environmental Health, Faculty of Health Sciences, University of Malta, Msida, Malta*

*^3^Metamaterials Unit, Faculty of Science, University of Malta, Msida, Malta*

[**vasilis.valdramidis@um.edu.mt*](mailto:*vasilis.valdramidis@um.edu.mt)

[**ruben.gatt@um.edu.mt*](mailto:*ruben.gatt@um.edu.mt)

**SEM images of coated filters**

The visual analysis of the SEM images indicates that, as expected, increasing the immersion time increases the thickness of the nanoparticle coating on the filter fibres. When comparing the same concentration and the same immersion time for Needlepunched and meltblown filters, it is evident that the Needlepunched filters are more heavily coated than the meltblown equivalent.

*SEM Images of ZnO nanoparticle coated Needlepunched filters*


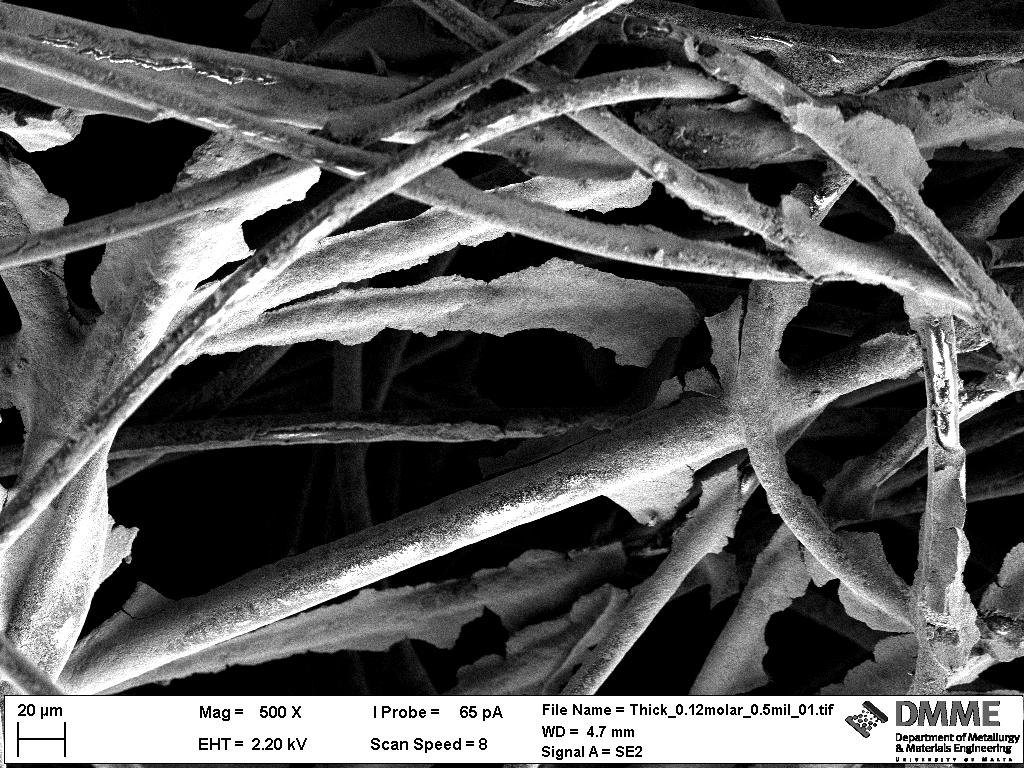

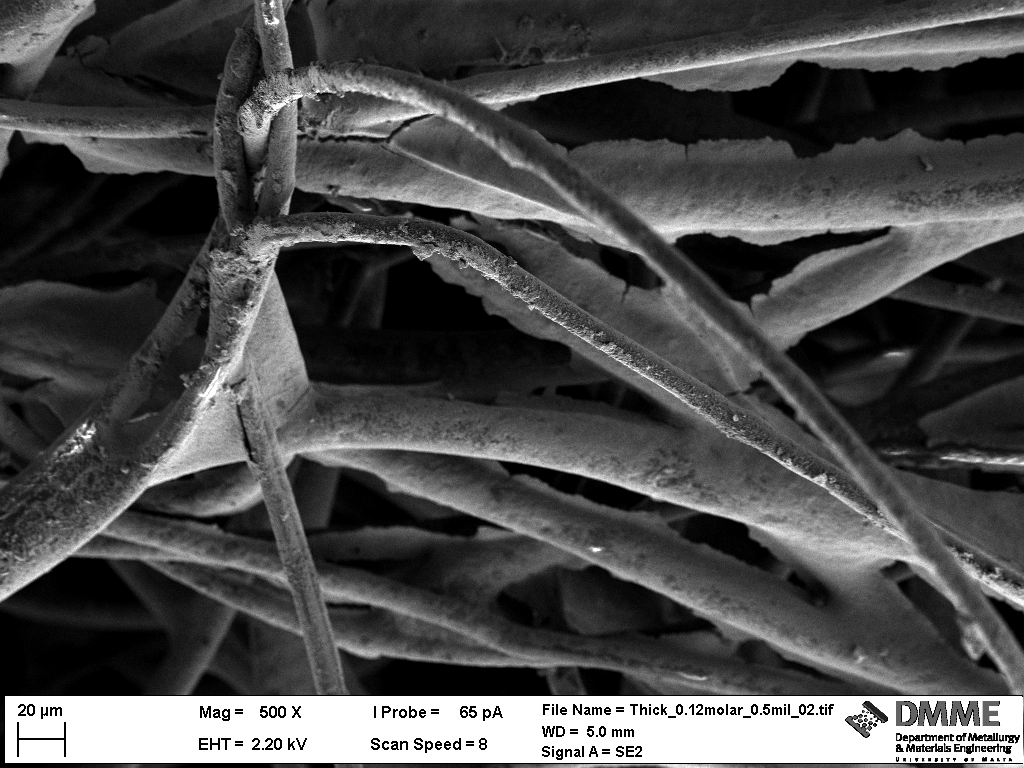


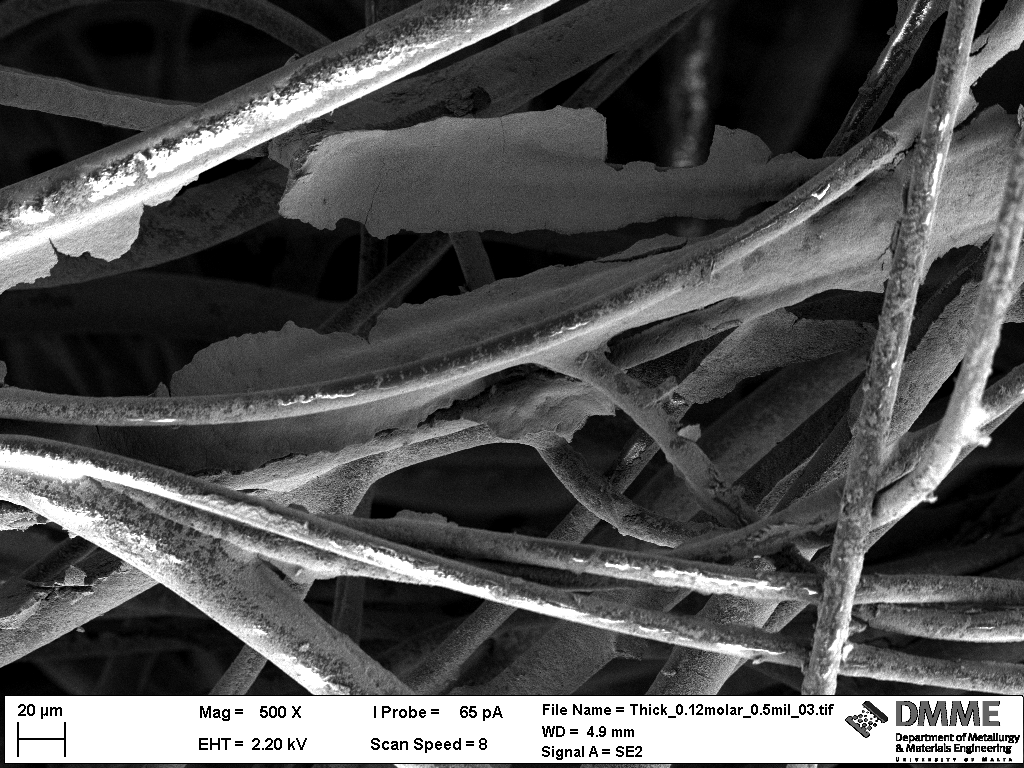

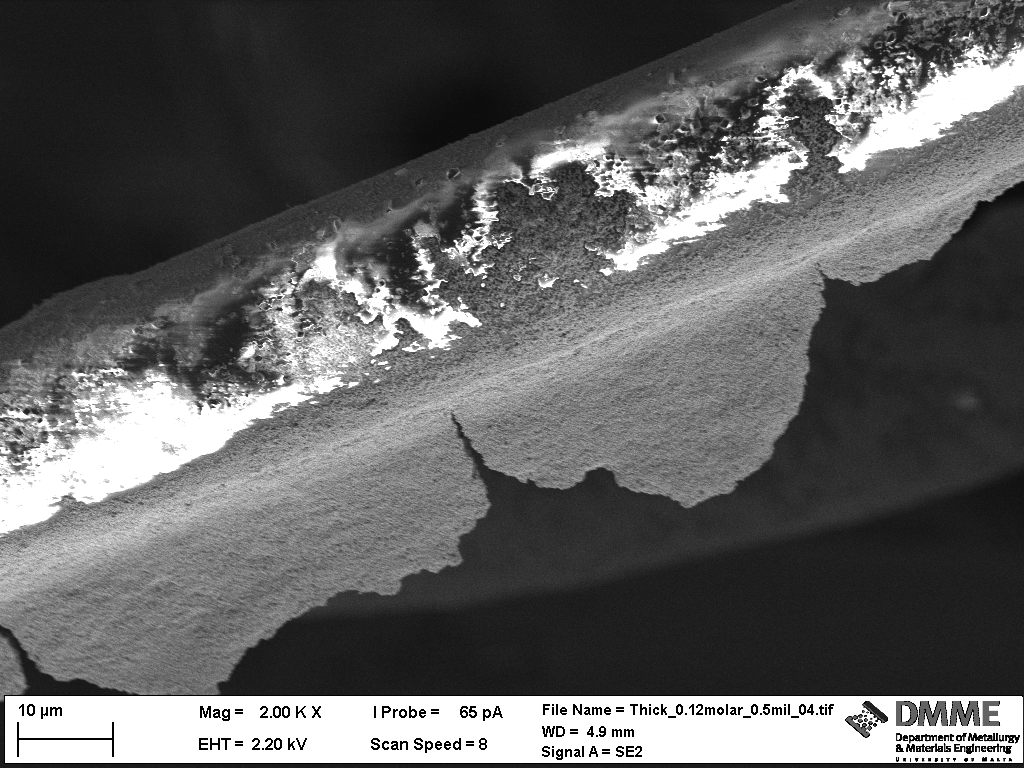


**Figure S1: Coating achieved using 0.12 molar solution with 0.5 minutes immersion time (Sample ID N_7_).**

*SEM Images of ZnO nanoparticle coated Meltblown filters*


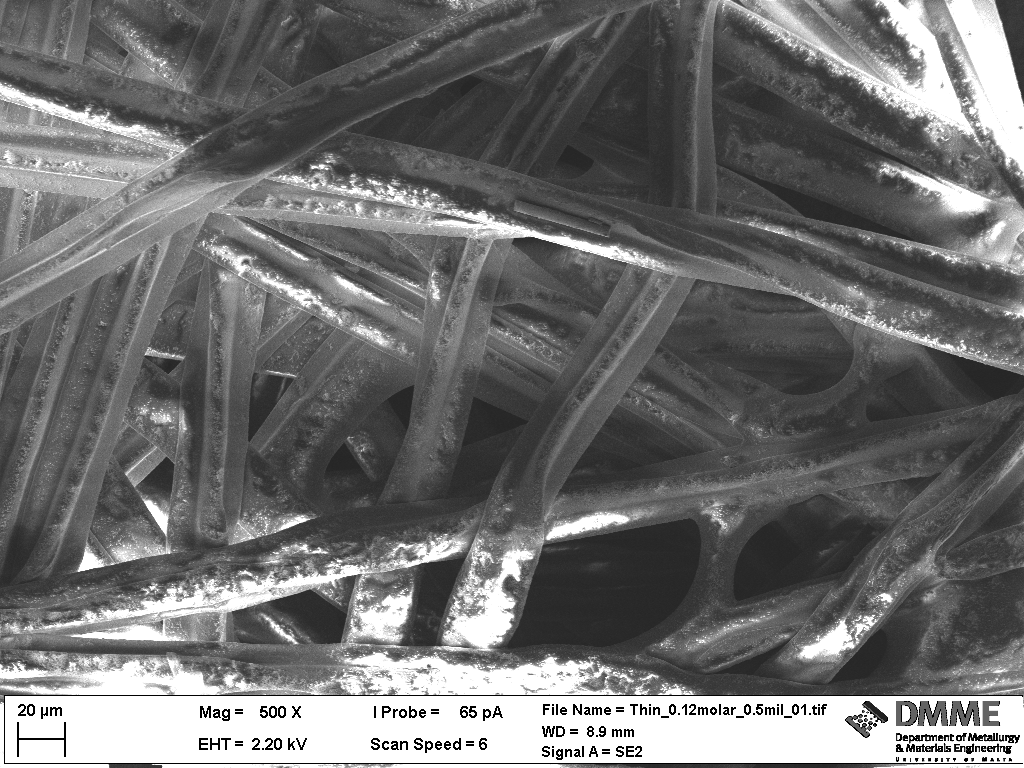

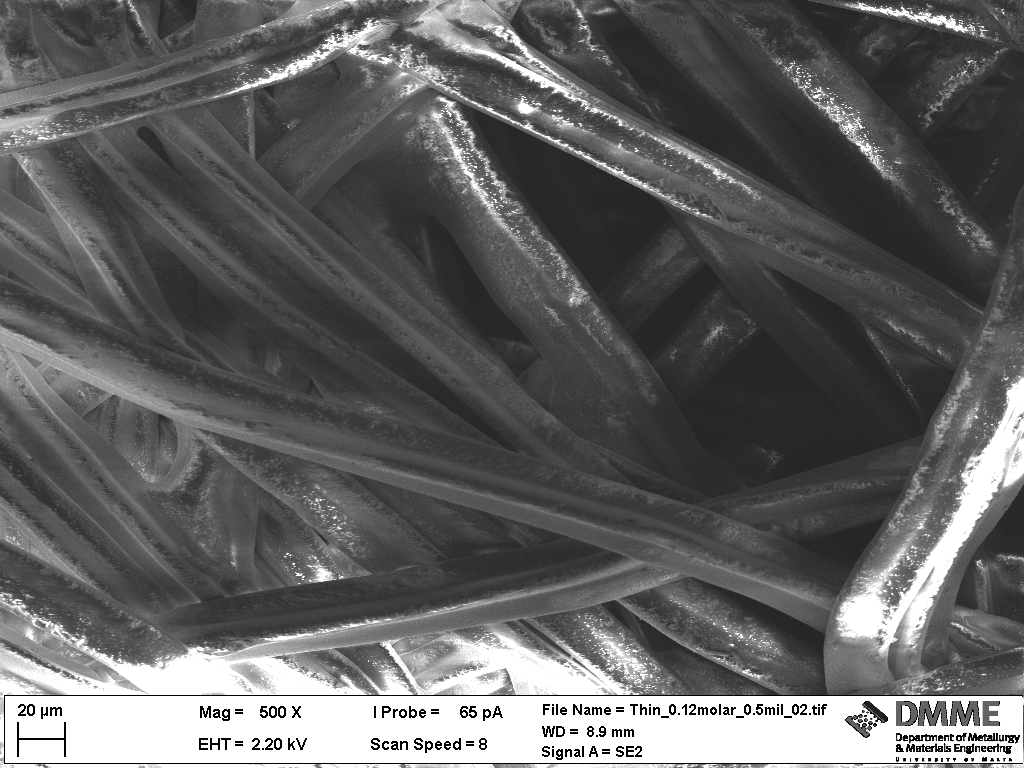


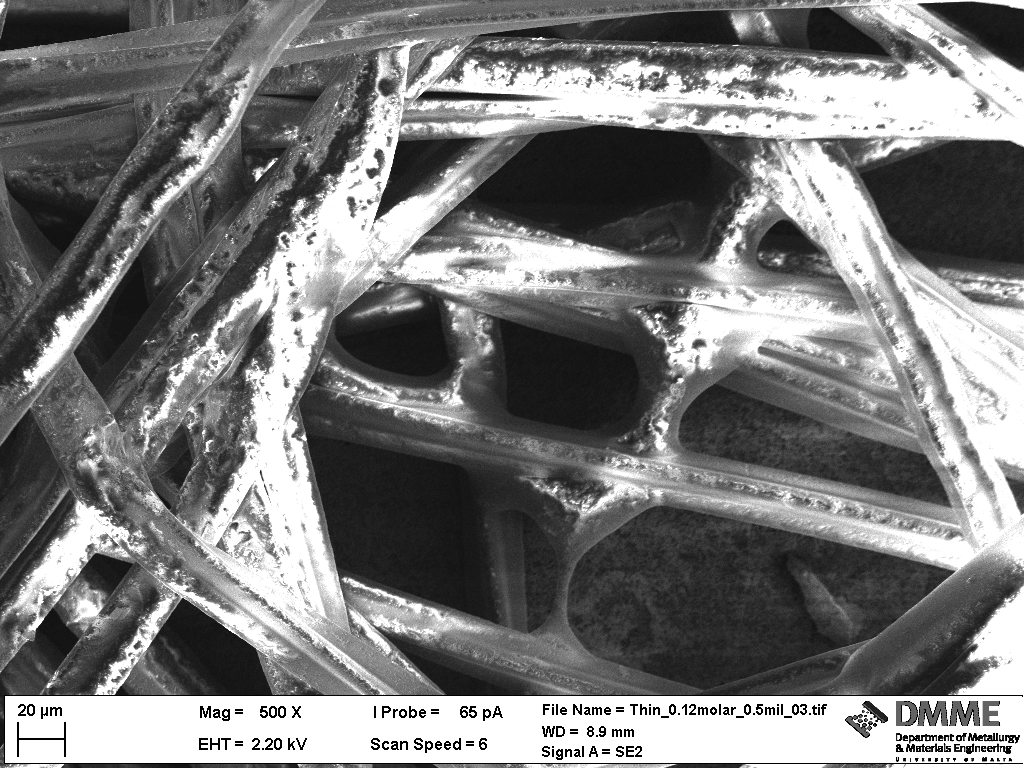


**Figure S2: Coating achieved using 0.12 molar solution with 0.5 minutes immersion time (Sample ID: M_7_).**


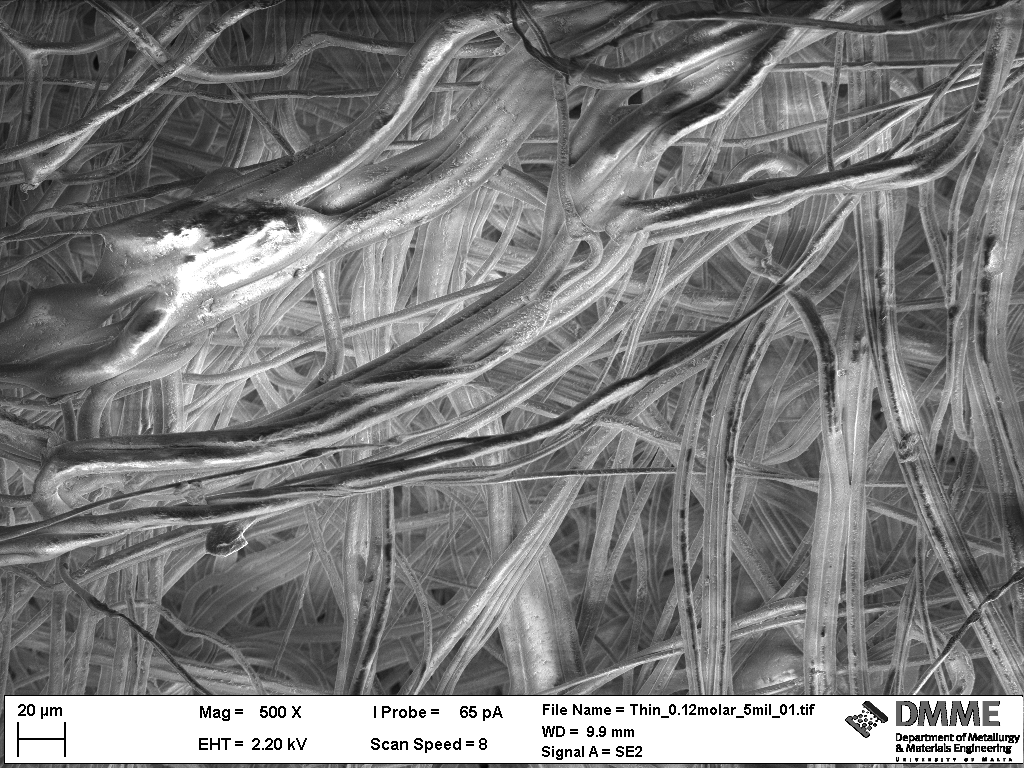

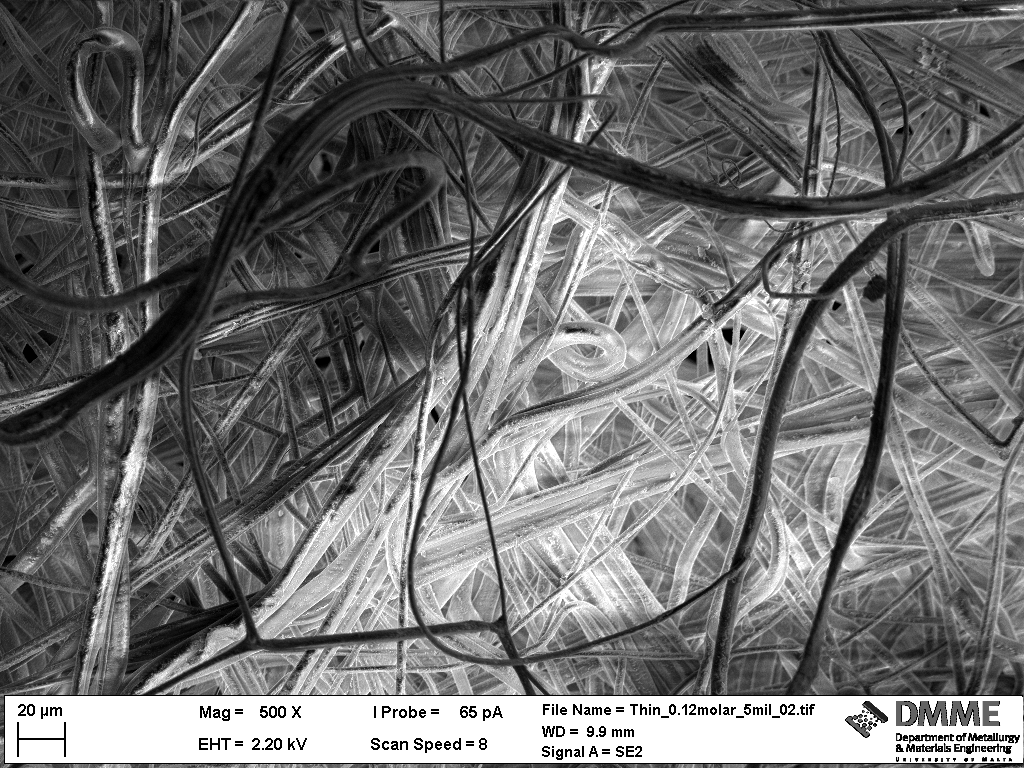


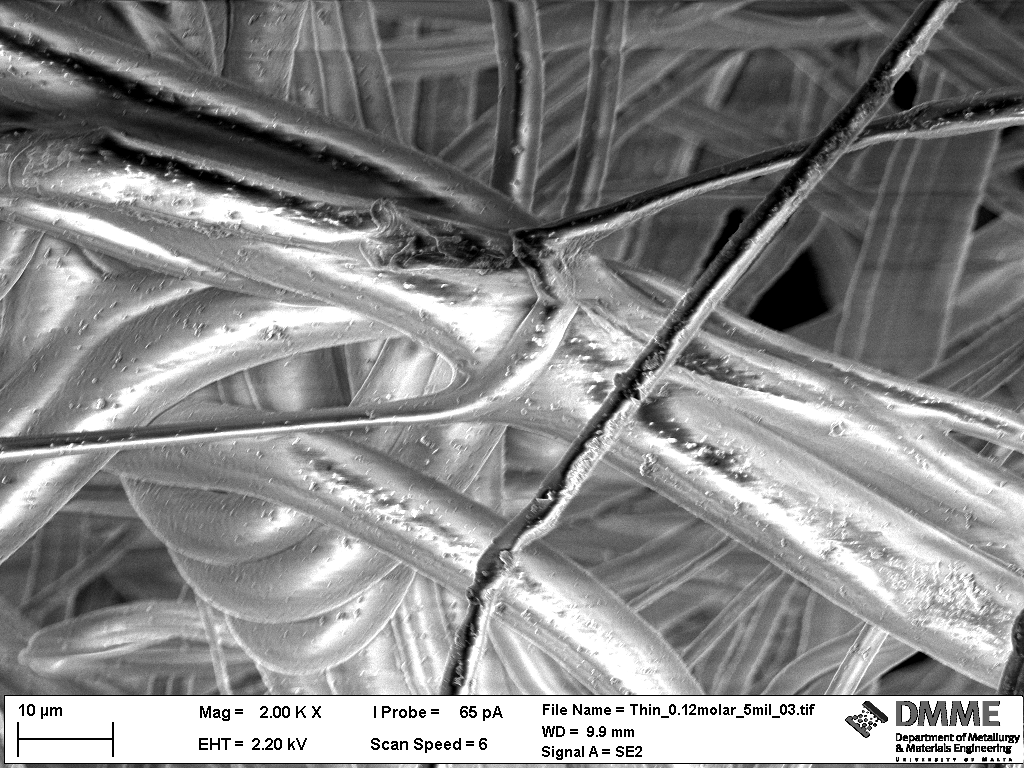


**Figure S3: Coating achieved using 0.12 molar solution with 5 minutes immersion time (Sample ID: M_8_)**

**Anti-fungal Activity of Coated Filter media**

A modification of the slide culture method was used to test the nanoparticles coated filters for growth inhibition. An overview of the experimental protocol is presented hereunder.

**Figure S5: Procedure followed for the coating of the needlepunched and the meltblown filters**

**Figure S6: Procedure followed for the assessment of the antifungal properties of the coated filters.**

**Figure S7: Example of results obtained (left photo: negative response, right photo: positive response).**

Hereudner the results of these experiments are presented with the corresponding replications

**Table S1: Replicated results of the binary responses showing gworth (+) and no growth (-) of *Penicillium expansum* on 0.012M ZnO coated filters. Each epxeriment was repeted in triplicate . Notation 1, 2, 3, refers to negative control samples at 0.5, 5 and 50 min, respectively.**

| **0.012M Zn** |  | **Sample ID** | **10^2^ s/mL** | **10^3^ s/mL** | **10^4^ s/mL** |
| --- | --- | --- | --- | --- | --- |
| *Needlepunched* | C | N_0_ | +++ | +++ | +++ |
|  | C- | N_1_,N_2_, N_3_ | +++ | +++ | +++ |
|  | 0.5 min | N_4_ | --- | --- | --- |
|  | 5 min | N_5_ | --- | --- | --- |
|  | 50 min | N_6_ | --- | --- | --- |
|  |  |  |  |  |  |
| **0.012M ZnO** |  | **Sample ID** | **10^2^ s/mL** | **10^3^ s/mL** | **10^4^ s/mL** |
| *Meltblown* | C | M_0_ | +++ | +++ | +++ |
|  | C- | M_1_,M_2_, M_3_ | +++ | +++ | +++ |
|  | 0.5 min | M_4_ | +++ | +++ | +++ |
|  | 5 min | M_5_ | --- | --- | +++ |
|  | 50 min | M_6_ | --- | --- | +++ |

**Table S2: Replicated results of the binary responses showing gworth (+) and no growth (-) of *Rhizopus stolonifer* on 0.012M ZnO coated filters. Each epxeriment was repeted in triplicate. Notation 1, 2, 3, refers to negative control samples at 0.5, 5 and 50 min, respectively.**

| **0.012M Zn** |  | **Sample ID** | **10^2^ s/mL** | **10^3^ s/mL** | **10^4^ s/mL** |
| --- | --- | --- | --- | --- | --- |
| *Needlepunched* | C | N_0_ | +++ | +++ | +++ |
|  | C- | N_1_,N_2_, N_3_ | +++ | +++ | +++ |
|  | 0.5 min | N_4_ | --- | +++ | +++ |
|  | 5 min | N_5_ | --- | +++ | +++ |
|  | 50 min | N_6_ | --- | --- | +++ |
|  |  |  |  |  |  |
| **0.012M ZnO** |  | **Sample ID** | **10^2^ s/mL** | **10^3^ s/mL** | **10^4^ s/mL** |
| *Meltblown* | C | M_0_ | +++ | +++ | +++ |
|  | C- | M_1_,M_2_, M_3_ | +++ | +++ | +++ |
|  | 0.5 min | M_4_ | +++ | +++ | +++ |
|  | 5 min | M_5_ | --- | --- | +++ |
|  | 50 min | M_6_ | --- | --- | --- |

**Table S3: Replicated results of the binary responses showing gworth (+) and no growth (-) of *Penicillium expansum* on 0.12M ZnO coated filters. Each epxeriment was repeted in triplicate. Notation 1, 2, 3, refers to negative control samples at 0.5, 5 and 50 min, respectively.**

| **0.12M Zn** |  | **Sample ID** | **10^2^ s/mL** | **10^3^ s/mL** |
| --- | --- | --- | --- | --- |
| *Needlepunched* | C | N_0_ | +++ | +++ |
|  | C- | N_1_,N_2_, N_3_ | +++ | +++ |
|  | 0.5 min | N_7_ | --- | --- |
|  | 5 min | N_8_ | --- | --- |
|  |  |  |  |  |
| **0.12M ZnO** |  | **Sample ID** | **10^2^ s/mL** | **10^3^ s/mL** |
| *Meltblown* | C | M_0_ | +++ | +++ |
|  | C- | M_1_,M_2_, M_3_ | +++ | +++ |
|  | 0.5 min | M_7_ | --- | --- |
|  | 5 min | M_8_ | --- | --- |

**Table S4: Replicated results of the binary responses gworth (+) and no growth**

**(-) of *Rhizopus stolonifer* on 0.12M ZnO coated filters. Each epxeriment was repeted in triplicate. Notation 1, 2, 3, refers to negative control samples at 0.5, 5 and 50 min, respectively.**

| **0.12M Zn** |  | **Sample ID** | **10^2^ s/mL** | **10^3^ s/mL** |
| --- | --- | --- | --- | --- |
| *Needlepunched* | C | N_0_ | +++ | +++ |
|  | C- | N_1_,N_2_, N_3_ | +++ | +++ |
|  | 0.5 min | N_7_ | --- | --- |
|  | 5 min | N_8_ | --- | --- |
|  |  |  |  |  |
| **0.12M ZnO** |  | **Sample ID** | **10^2^ s/mL** | **10^3^ s/mL** |
| *Meltblown* | C | M_0_ | +++ | +++ |
|  | C- | M_1_,M_2_, M_3_ | +++ | +++ |
|  | 0.5 min | M_7_ | --- | --- |
|  | 5 min | M_8_ | --- | --- |

From the above results it is evident that that *P. expansum* was the most inhibited on the 0.012M ZnO coated filters and could be even inhibited when filters were coated for only 30 sec in the case of needlepunched filters. *R. stolonifer* showed less sensitivity when compared with *P. expansum*. The longer the coating time, the more effective the inhibition for all the tested fungi. The 0.12M concentration of ZnO inhibited all the studied fungi even for the shortest coating time of 30 sec.

**Stress-Strain Graphs**


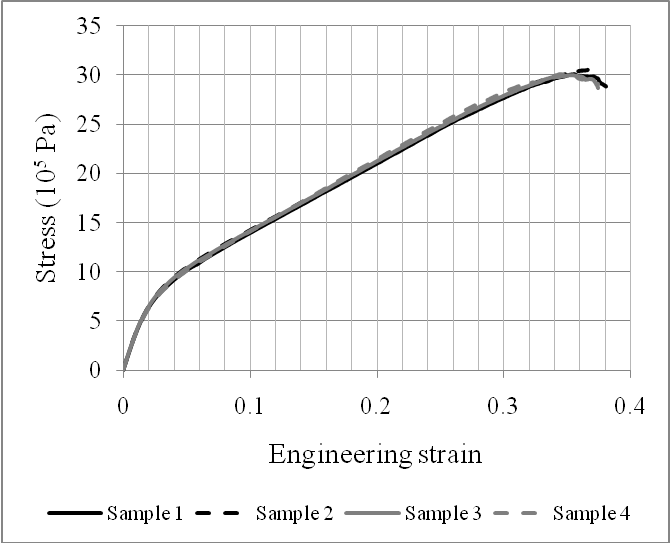

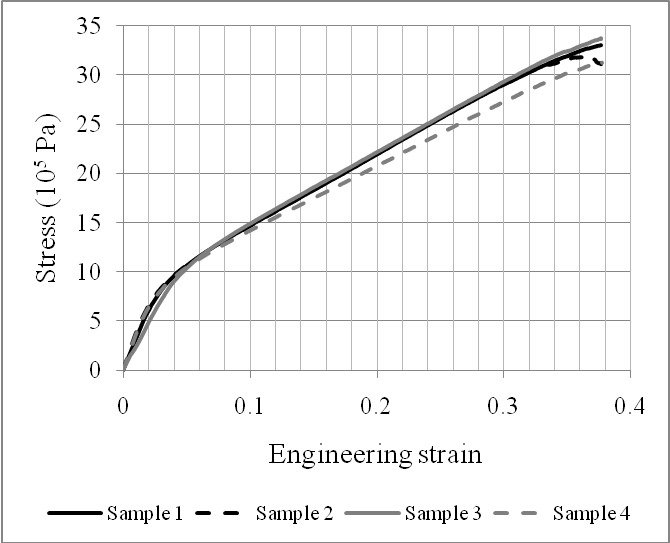


Graph 1 – Sample ID: N_0_ Graph 2 – Sample ID: N_1_


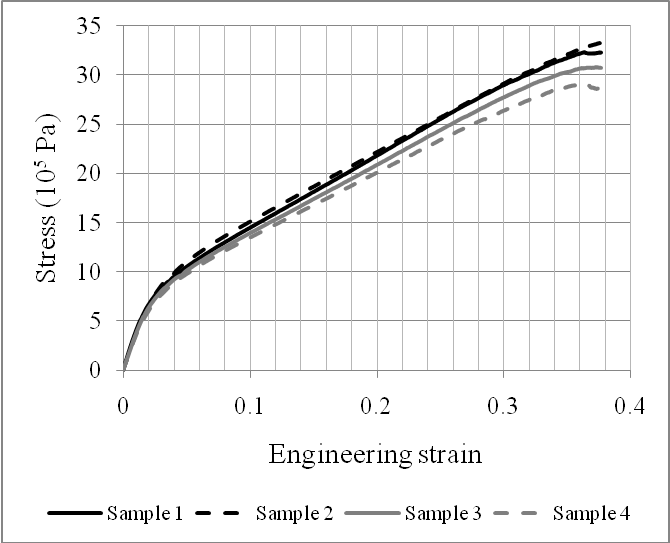

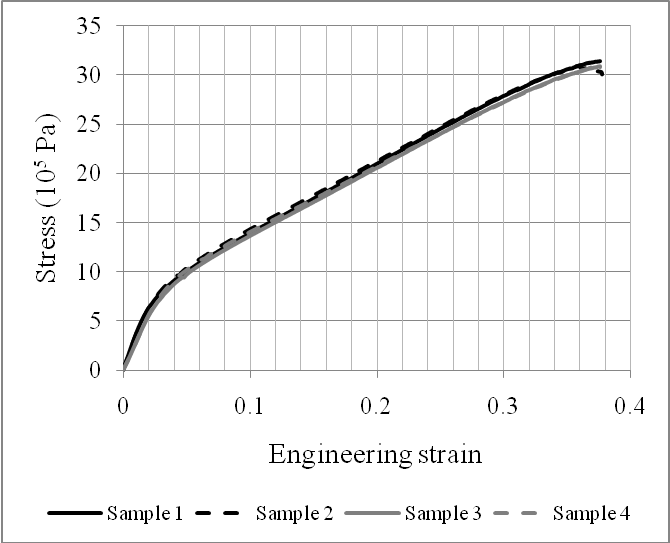


Graph 3 – Sample ID: N_2_ Graph 4 – Sample ID: N_3_


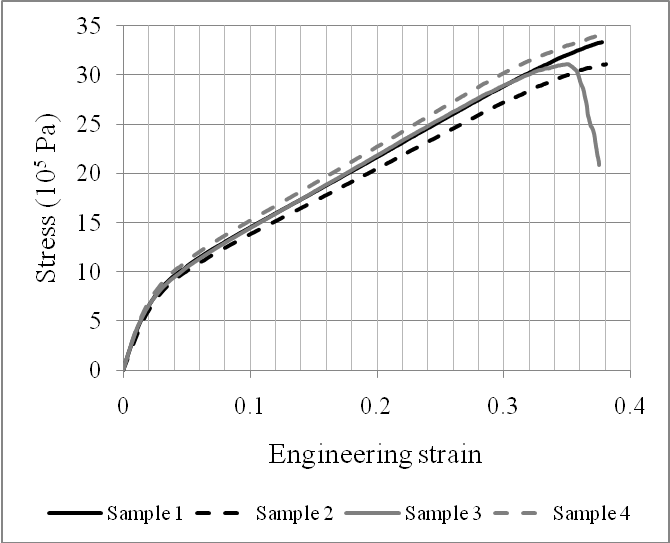

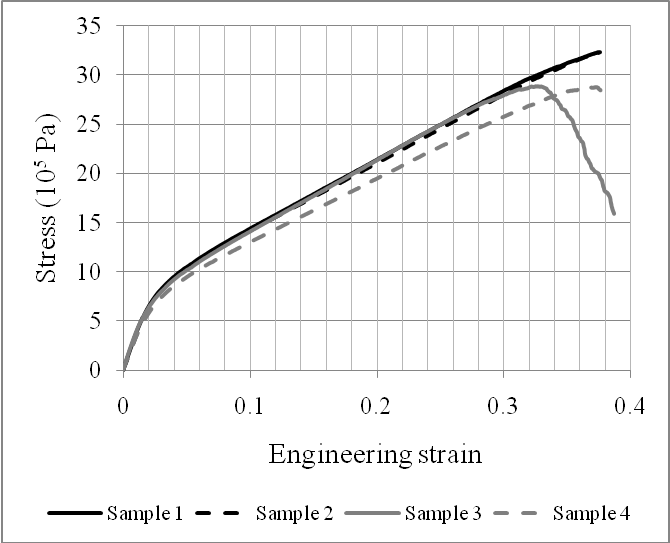


Graph 5 – Sample ID: N_4_ Graph 6 – Sample ID: N_5_

**Figure S8: Stress-strain curves for filter samples N_0_ to N_5_.**


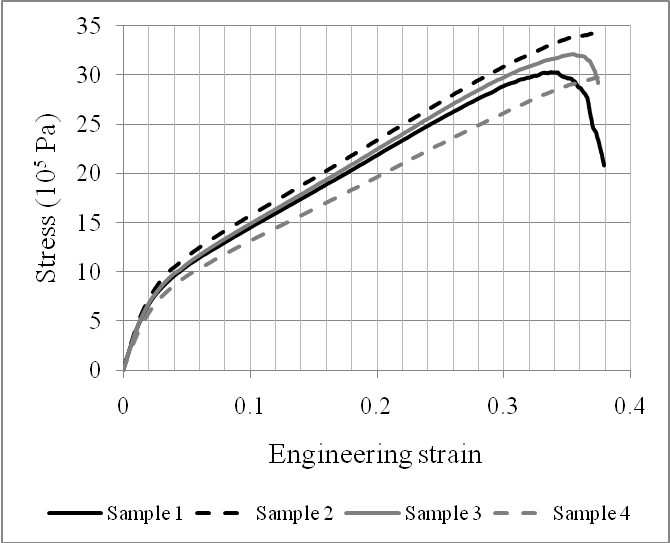

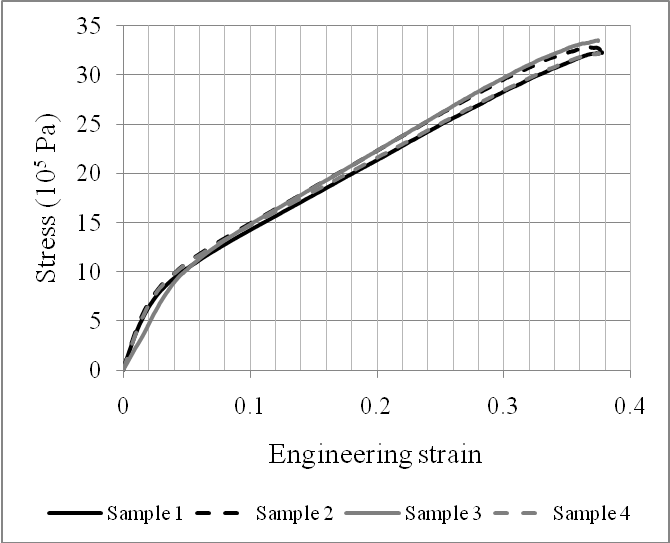


Graph 7 – Sample ID: N_6_ Graph 8 – Sample ID: N_7_


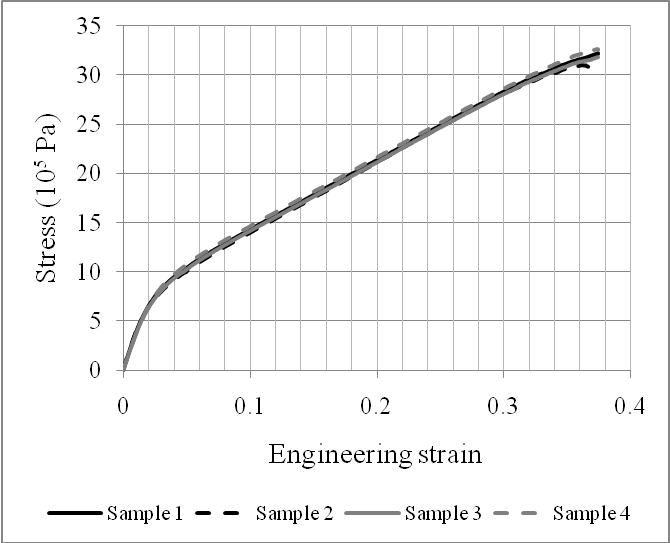

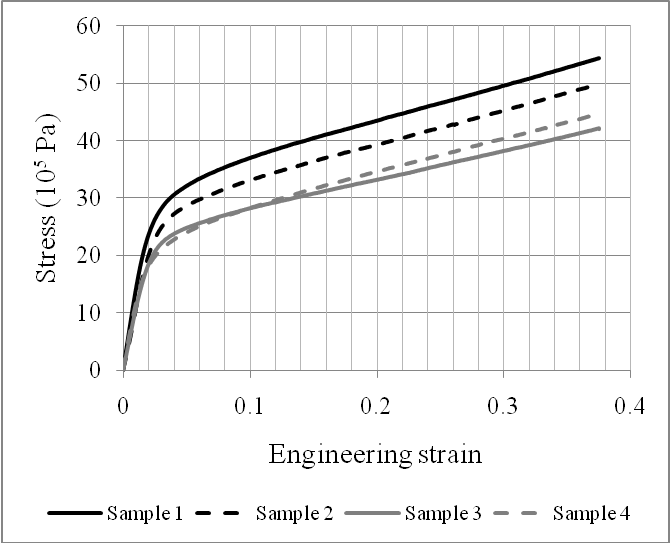


Sample ID: Graph 9 – N_8_ Graph 10 – Sample ID: M_0_


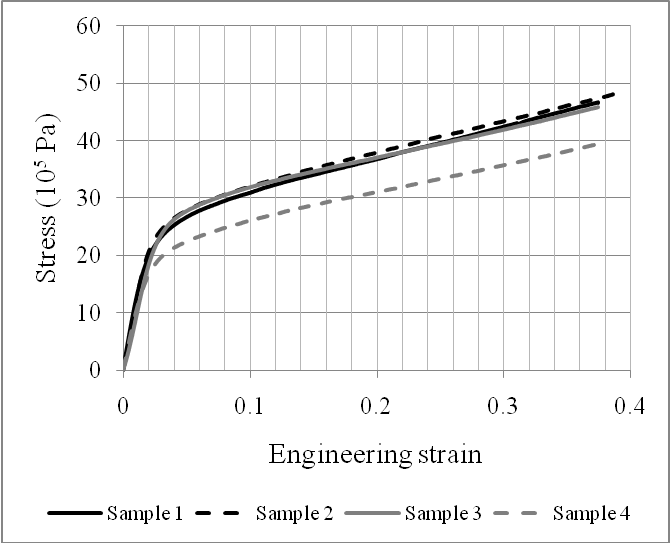

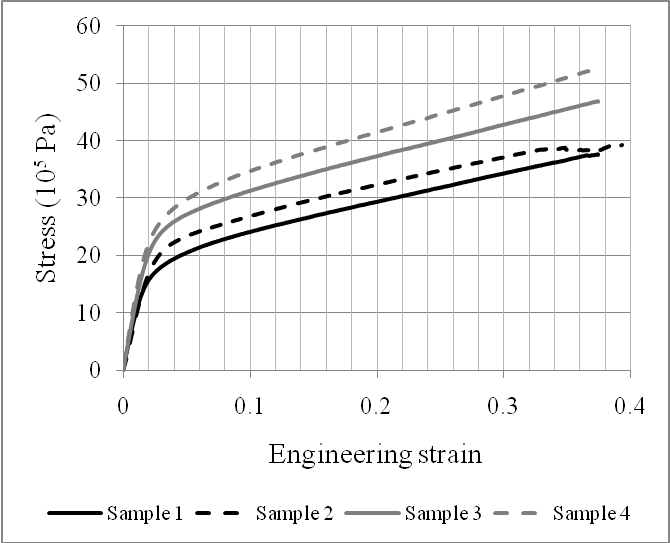


Graph 11 – Sample ID: M_1_ Graph 12 – Sample ID: M_2_

**Figure S9: Stress-strain curves for filter samples N_6_ to N_8_ and M_0_ to M_2_.**


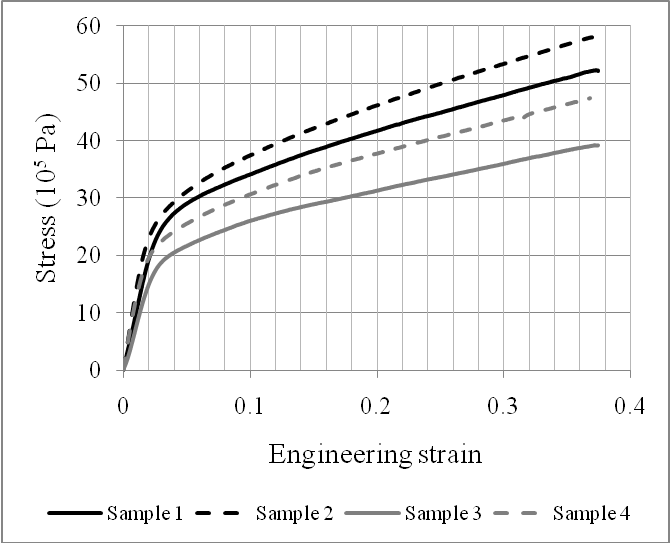

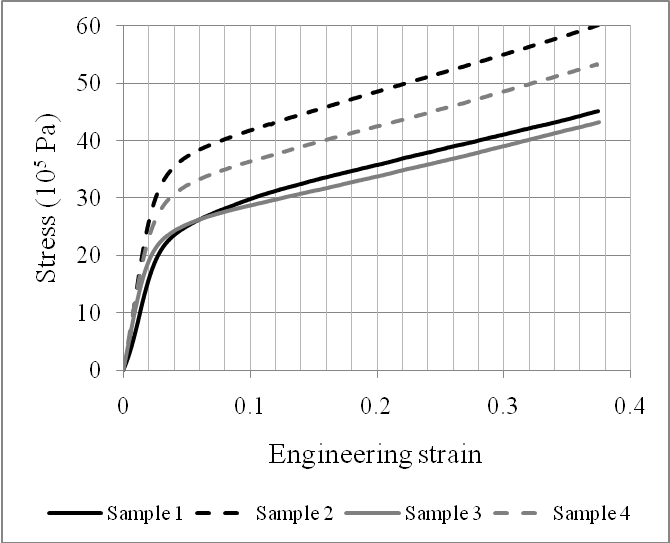


Graph 13 – Sample ID: M_3_ Graph 14 – Sample ID: M_4_


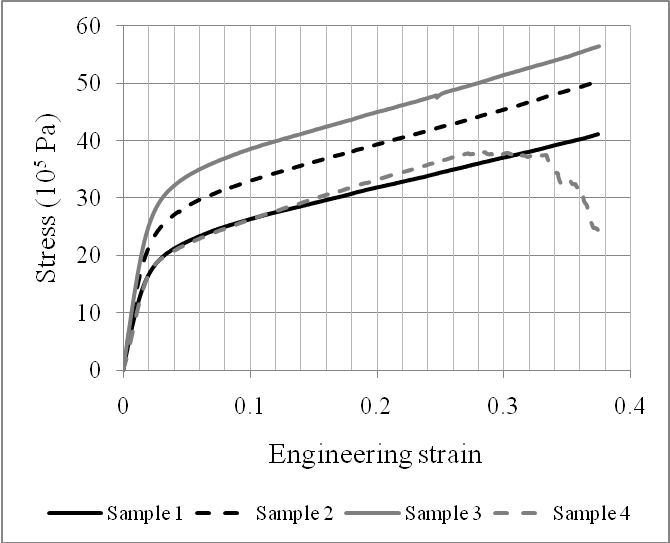

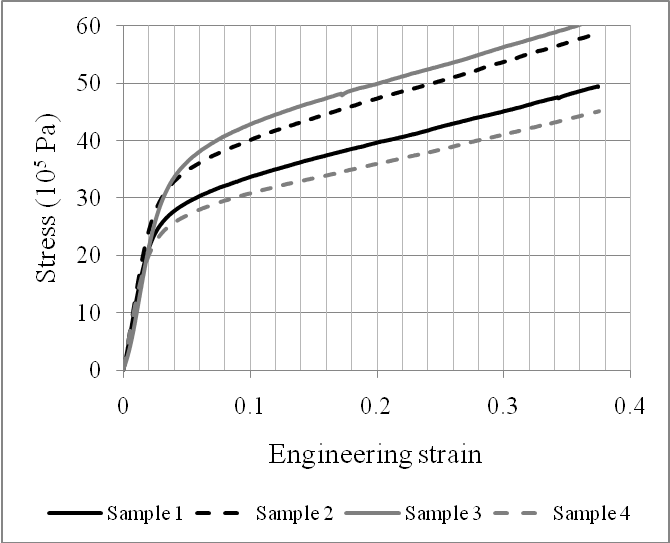


Graph 15 – Sample ID: M_5_ Graph 16 – Sample ID: M_6_


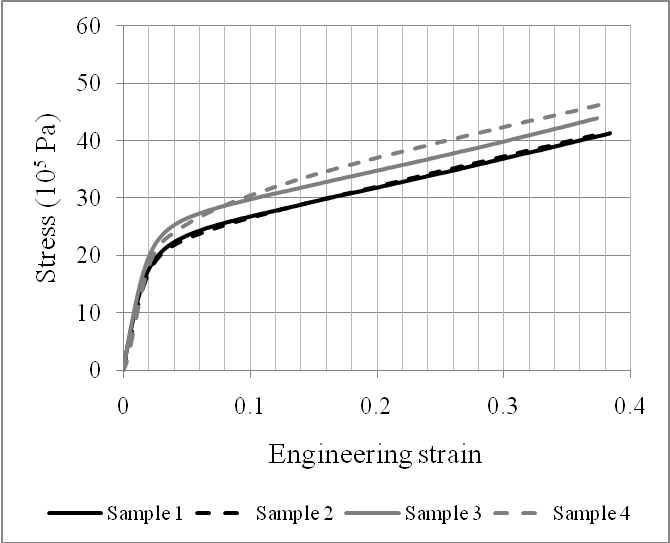

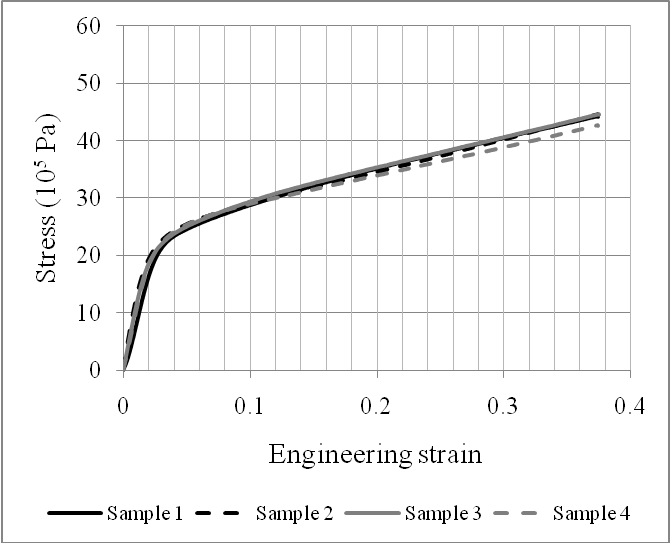


Graph 17 – Sample ID: M_7_ Graph 18 – Sample ID: M_8_

**Figure S10: Stress-strain curves for filter samples M_3_ to M_8_.**

From the stress-strain curves presented above, it is evident that both in the case of the Needlepunched filters and Meltblown filters, the initial part of the stress-strain curve (low strains) is quite similar for the 4 repeats done for each filter treatment. However, in the case of the Meltblown filters, the repeatability of the tests is low at high strains, particularly for those that were not coated with nanoparticles.

From a comparison of the stress-strain curves of the Needlepunched and Meltblown filters one notes that the yield point for the Needlepunched filters is obtained at slightly higher strains. However, in all cases tested here, the Meltblown filters have a higher yield strength and Ultimate strength.
